# RIN3 Is a Negative Regulator of Mast Cell Responses to SCF

**DOI:** 10.1371/journal.pone.0049615

**Published:** 2012-11-20

**Authors:** Christine Janson, Noriyuki Kasahara, George C. Prendergast, John Colicelli

**Affiliations:** 1 Molecular Biology Institute, Jonsson Comprehensive Cancer Center, Department of Biological Chemistry, David Geffen School of Medicine, University of California Los Angeles, Los Angeles, California, United States of America; 2 Department of Medicine, David Geffen School of Medicine, University of California Los Angeles, Los Angeles, California, United States of America; 3 Lankenau Institute for Medical Research, Wynnewood, Pennsylvania, United of States of America; Hungarian Academy of Sciences, Hungary

## Abstract

Stimulation of the receptor tyrosine kinase KIT by Stem Cell Factor (SCF) triggers activation of RAS and its downstream effectors. Proper KIT activation is essential for the maturation, survival and proliferation of mast cells. In addition, SCF activation of KIT is critical for recruiting mast cells to sites of infection or injury, where they release a mix of pro-inflammatory substances. RIN3, a RAS effector and RAB5-directed guanine nucleotide exchange factor (GEF), is highly expressed and enriched in human mast cells. SCF treatment of mast cells increased the amount of GTP-bound RAB5, and the degree of RAB5 activation correlated with the expression level of RIN3. At the same time, SCF caused the dissociation of a pre-formed complex of RIN3 with BIN2, a membrane bending protein implicated in endocytosis. Silencing of RIN3 increased the rate of SCF-induced KIT internalization, while persistent RIN3 over-expression led to KIT down regulation. These observations strongly support a role for RIN3 in coordinating the early steps of KIT endocytosis. Importantly, RIN3 also functioned as an inhibitor of mast cell migration toward SCF. Finally, we demonstrate that elevated RIN3 levels sensitize mastocytosis cells to treatment with a KIT tyrosine kinase inhibitor, suggesting the value of a two-pronged inhibitor approach for this difficult to treat malignancy. These findings directly connect KIT activation with a mast cell-specific RAS effector that regulates the cellular response to SCF and provide new insight for the development of more effective mastocytosis treatments.

## Introduction

Mast cells are critical for allergic inflammatory responses, including type I hypersensitivity, anaphylaxis, asthma, and arthritis (reviewed in [1], [2]). The most abundant tyrosine kinase receptor on a mast cell surface is KIT (c-KIT, CD117) (reviewed in [3]–[5]). Signaling is induced by the binding of its ligand, Stem Cell Factor (SCF), and is required for mast cell maturation, proliferation and migration. SCF also enhances mast cell responses leading to allergic airway inflammation and hyperreactivity [6]. KIT is expressed in germ cells and hematopoietic stem/progenitor cells, but among mature somatic cells it is restricted primarily to mast cells and melanocytes [7]. Gain of function mutations in KIT are causative in hyper-proliferative pathologies originating from these cells, including mast cell-derived mastocytosis, a family of diseases characterized by mast cell hyper-proliferation [8]. The spectrum of these diseases ranges from asymptomatic, indolent systemic mastocytosis to malignant, aggressive mast cell leukemia [9].

The endocytosis of receptor tyrosine kinases (RTKs), such as KIT, begins with ligand-induced receptor dimerization and transphosphorylation. This leads to engagement of downstream signal transduction pathways, most notably those mediated by RAS family GTPases, that drive the cell’s immediate and long-term response to stimulation. Activated RTKs are typically internalized through an endocytosis mechanism mediated by clathrin and membrane deforming proteins including those in the amphiphysin family of BAR domain proteins [10]–[12]. Internalized RTKs may continue to send downstream signals from early endosomes. Endocytosed receptors ultimately face one of two fates: recycling and replacement on the plasma membrane or degradation via the proteasome or lysosome. The RAB5 family of GTPases mediate early steps in endocytosis including early endosome fusion [13]–[16] and play an important role in determining the fate of internalized receptors [17], [18].

RIN3 is a member of the RIN family of RAS effectors [19], all of which have a guanine nucleotide exchange factor (GEF) domain with specificity for RAB5 family GTPases as well as a RAS association (RA) domain and an SH2 domain. The most extensively studied member of the RIN family is RIN1, which directly controls the signaling and stability of EGFR and other receptor tyrosine kinases [20]–[22] and may indirectly influence the endocytosis of other receptors [23]–[26]. In epithelial cells, growth factor stimulation of RTKs leads to activation of RAS effectors such as RIN1, which in turn activates RAB5 proteins and promotes RTK down regulation.

In this study we show that RIN3 displays a tissue-specific expression pattern, with highest levels restricted to mast cells. RIN3 was an effective promoter of endogenous RAB5 activation in human mast cells. RIN3 silencing accelerated the rate of KIT internalization following SCF stimulation, while down regulation of KIT was significantly enhanced by RIN3 over-expression. The ability of mast cells to migrate toward SCF, which requires KIT recycling and prolonged signaling, was inversely correlated with RIN3 expression. Importantly, RIN3 over-expression sensitized a mastocytosis cell line to treatment with the KIT inhibitor imatinib. By regulating KIT response and stability, RIN3 may play a key role in basic mast cell functions as well as pathologies involving mast cell mediated chronic inflammation and mast cell hyperproliferation.

## Results

### RIN3 is Highly Enriched in Mast Cells

**Figure 1:**
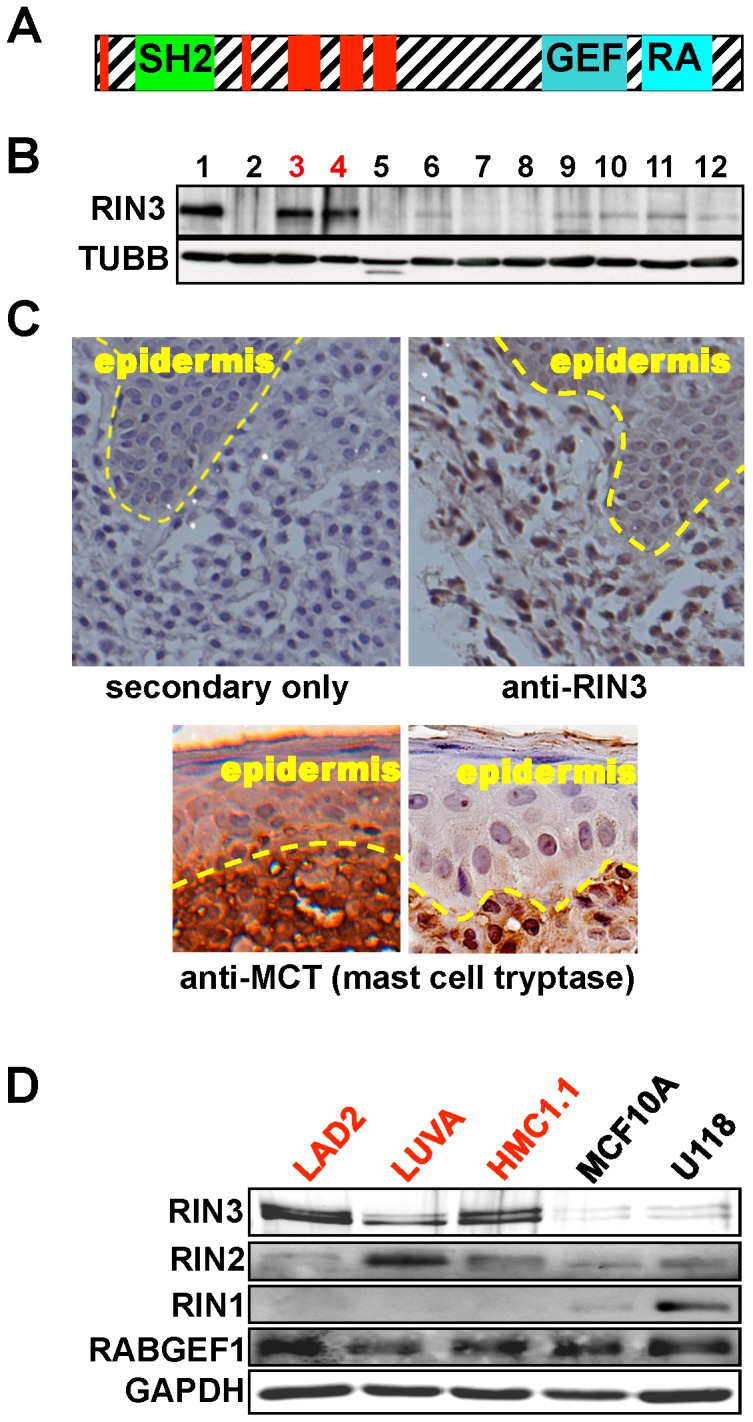
RIN3 is highly expressed and enriched in human mast cells. (A) The domain structure of RIN3. Red = proline-rich motifs. (B) Immunoblot analysis of RIN3 expression in human cell lines. 1:NIH3T3 w/RIN3 (+ctr), 2:NIH3T3 (−ctr), 3:HMC1 (mast), 4:LAD2 (mast), 5:THP1 (macrophage), 6:SaOs2 (osteoclast), 7:RAMOS (B cell), 8:K562 (myoblast), 9:Jurkat (T cell), 10: MCF10A (epithelial), 11:IMR90 (fibroblast), 12:U118 (glioblastoma). RIN3 appears as a double or single band depending on exposure time of the immunoblot. TUBB = β-tubulin. (C) Human mastocytosis sections stained using immunohistochemistry for RIN3 or secondary only as a control. Sections stained with anti-mast cell tryptase are shown to identify the infiltrating mast cells. (D) Lysates from three human mast cell lines (LAD2, LUVA, HMC1.1), an epithelial cell line (MCF10A), and a neuronal cell line (U118) were run on an SDS-PAGE gel and immunoblotted for RIN family members (RIN1/2/3) as well as RABGEF1, another GEF for RAB5.

The domain structure of RIN3 (Fig. 1A) suggests functional similarity with RIN1, a known regulator of RTK endocytosis in epithelial cells and neurons [23], [24], [27], [28]. This led us to hypothesize that RIN3 may serve as a regulator of RTK endocytosis in a restricted and perhaps distinct set of cell types. We therefore examined an expression database (BioGPS) and found that human RIN3 mRNA was most highly expressed in CD14^+^ monocytes (51 fold above median for all tissues) and mouse Rin3 expression was highest in mast cells (24 fold above median for all tissues). To examine if this relatively restricted expression pattern was also true at the protein level, a panel of human cell lines derived from various lineages was immunoblotted for endogenous RIN3. Two mast cell lines, HMC and LAD2, showed by far the highest expression of RIN3 protein (Fig. 1B). Representative macrophage and osteoclast cells, which, like mast cells, are derived from bone marrow, showed low or undetectable RIN3 protein. This was also true for representative B cell, myeloblast, T cell, fibroblast and gliobastoma lines.

RIN3 was detected by immunohistochemistry in tissue samples from mastocytosis patients (Fig. 1C). While RIN3 expression was easily detected in the mast cells, bordering epidermal cells showed signal intensity about equal to tissue stained with secondary antibody only. These results confirm that RIN3 expression is characteristic of primary human mastocytosis cells, and did not result from the generation of mast cell lines.

We next compared the expression of RIN3 to other members of the RIN family of proteins (Fig. 1D). Expression of RIN3 was high in all three mast cell lines examined (LAD2, LUVA, and HMC1.1) with little expression in representative epithelial and glioblastoma cell lines (Fig. 1D). RIN1, the best characterized of the RIN paralog family, showed highest expression in glioblastoma cells and low but detectable expression in epithelial cells but was undetectable in the mast cell lines. RIN2 expression was detected in all cell types with relatively high expression in the LUVA mast cell line.

RIN3 is a GEF for RAB5 GTPases. We therefore compared the expression profile of RIN3 to that of RABGEF1 (Rabex-5), a RAB5-targeted GEF known to play a role in mast cell function. RABGEF1 promotes internalization and affects the downstream signaling of both FcεRI, the high affinity IgE receptor, and KIT in mast cells [29], [30]. RABGEF1 also influences the endocytosis of RTKs in other cell types [31]. We found RABGEF1 expression in all cell lines probed (Fig. 1D), as expected based on its reported expression in multiple cell types (BioGPS.org and [32]).

The protein and mRNA data show that RIN3 expression is highly skewed, with notably elevated levels in mast cells. This restricted expression profile contrasts with what was seen for RIN1, RIN2 and the more distantly related RABGEF1, which show quite different tissue distribution biases or are widely expressed. Taken together, these findings suggest that RIN3 makes a unique contribution to mast cell function.

### RIN3 Interacts with Endogenous BIN2 in Mast Cells

**Figure 2:**
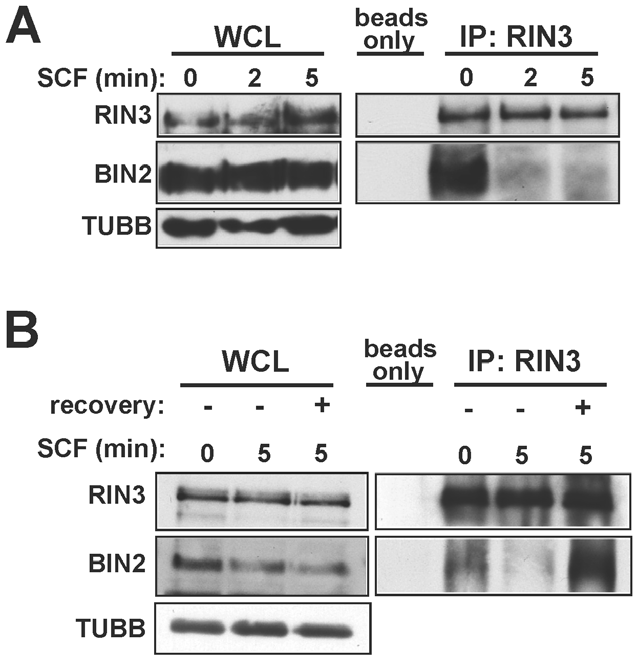
RIN3 interacts with endogenous BIN2. (A) Cells were stimulated with 100 ng/ml SCF for indicated time points and lysates were immuno-precipitated with anti-RIN3 or beads alone (ctrl). Whole cell lysate (WCL) and IP samples were immunoblotted for RIN3 and BIN2. The immunoblots shown are representative of two independent experiments. At five minutes post-stimulation the amount of BIN2 precipitated was reduced by 81±6% compared to unstimulated (ImageJ), p<0.05. (B) Cells were stimulated with 100 ng/ml SCF for 0 or 5 minutes, then lysed immediately or resuspended in SCF free medium to recover for 4 hours at 37°C before lysis. Lysates were immunoprecipitated and immunoblotted as in A.

Previous studies [19] reported that RIN3 interacts with BIN1, a BAR domain protein that binds to lipid membranes and induces bending associated with trafficking events [33], [34]. Endogenous BIN1 protein was below the level of detection in LAD2 cells. A paralog BAR domain protein, BIN2, is more highly expressed in hematopoietic cells compared to BIN1 [35]; therefore, we evaluated RIN3 binding to BIN2 in LAD2 cells. Cells were stimulated with 100 ng/mL SCF, and RIN3 was immunoprecipitated from cell lysates. BIN2 was bound to RIN3 in unstimulated cells, but this interaction was lost as early as 2 minutes after stimulation with SCF (Fig. 2A). This result indicates that RIN3 and BIN2 are normally connected to each other, either directly or as part of a complex, in resting mast cells. But soon after SCF treatment, and concurrent with KIT stimulation and internalization, RIN3 and BIN2 appear to dissociate from each other.

We next examined whether the association of BIN2 and RIN3 reflects the normal physiological state of resting mast cells by looking for the re-association of BIN2 and RIN3. LAD2 cells were stimulated with SCF to disrupt the RIN3::BIN2 complex and then allowed to recover in SCF-free medium. Four hours after the return of cells to SCF-free medium, RIN3 and BIN2 had re-associated (Fig 2B). This result suggests that RIN3 and BIN2 are part of a shared complex in resting mast cells, that stimulation of KIT leads to disruption of this complex and release of BIN2, and that the complex re-forms upon return to the resting state.

### RIN3 Negatively Regulates KIT Internalization

**Figure 3:**
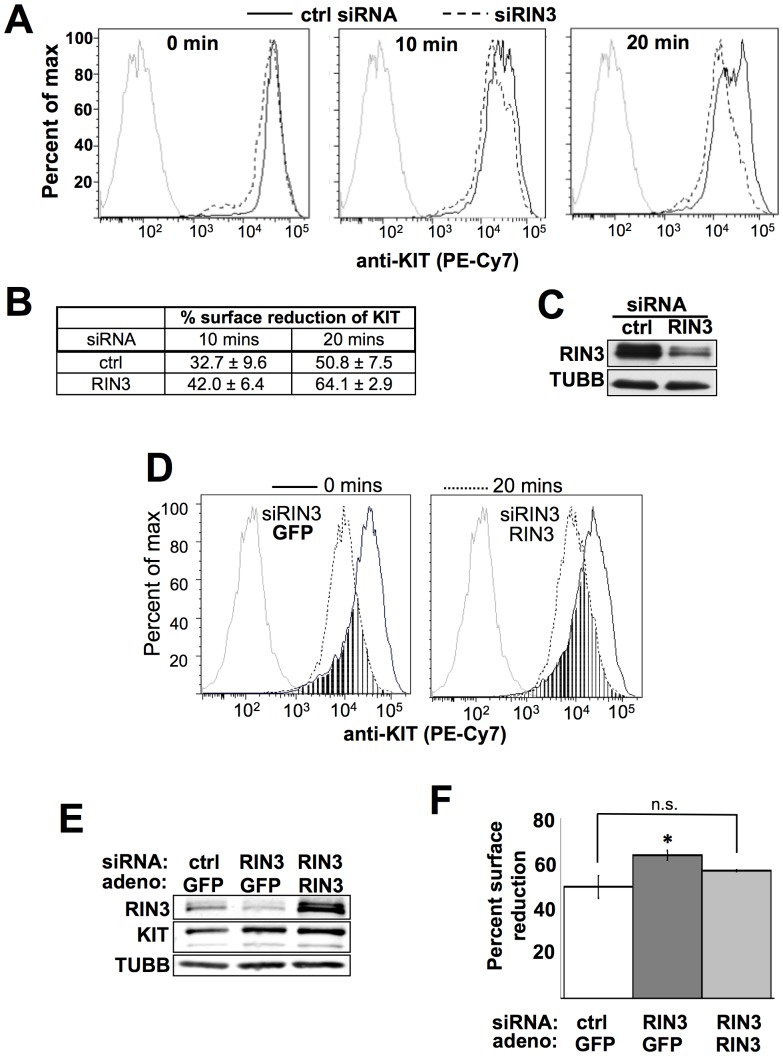
RIN3 negatively regulates the internalization of KIT post SCF stimulation. (A) Cells transfected with control (solid) or RIN3 siRNA (dashed) were stimulated with 5 ng/ml SCF for indicated time points. Cells were stained for surface intensity of KIT and analyzed by flow cytometry. Gray line indicates unstained control. (B) Table shows the percent surface reduction of KIT from three independent flow cytometry experiments. Surface reduction at 20 minutes post stimulation is significantly increased in RIN3 knock down cells, p<0.05. (C) Immunoblot indicating the level of RIN3 knockdown. (D) Cells were transfected with RIN3 siRNA and then infected with GFP (siRNA/GFP) or RIN3 (siRIN3/RIN3) adenovirus. Surface intensity of KIT was determined by flow cytometry at 0 mins (solid) and 20 mins (dotted) post stimulation. Percent reduction: ctrl/GFP (not shown): 59%, siRIN3/GFP: 67%, siRIN3/RIN3∶57%. (E) Immunoblot for RIN3 and KIT levels in transfected/infected cells. (F) Graph represents percent surface reduction at 20 minutes post stimulation from two independent experiments. Percent reduction of ctrl/GFP versus siRIN3/RIN3 is not significant (p>0.34). Percent reduction siRIN3/GFP versus siRIN3/siRIN3 is significant (p<0.05).

RIN1 is known to regulate the endocytosis and downstream signaling of receptor tyrosine kinases (RTKs) in epithelial cells [23], [36]. We hypothesized that RIN3 may be playing a role in regulating KIT, the Stem Cell Factor (SCF) receptor and most abundant RTK found on mast cells. To test our hypothesis, we measured the internalization of KIT following stimulation with SCF. We chose to perform these experiments in LAD2 cells, which express wild type KIT and are highly dependent on SCF for proliferation in culture [37]. RIN3 was silenced using a targeted siRNA. We observed a consistent decrease in RIN3 protein levels compared to control siRNA transfected cells, which were indistinguishable from mock infected cells (Fig. S1A). Control and RIN3 silenced LAD2 cells were stimulated with SCF at 5 ng/mL, a relatively low but still physiological concentration that facilitates measurement of early events after KIT activation. Stimulated mast cells were then analyzed for cell surface KIT by flow cytometry. RIN3 silencing did not influence the basal level of detectable KIT prior to SCF treatment (Fig. 3A, left), but by 10 minutes post stimulation the cells with reduced RIN3 expression showed a marked decrease in cells exhibiting high intensity surface KIT compared to control cells (Fig. 3A, middle). This difference was even more pronounced at 20 minutes post stimulation, when RIN3 silenced cells had a well defined, low staining intensity peak and little overlap with the high intensity peak of control cells (Fig. 3A, right). At 20 minutes post stimulation knock down cells have finished internalization of the receptor while a population within the control cells still had high levels of surface KIT. Cells with reduced RIN3 have a significantly greater percent of KIT surface reduction 20 minutes post stimulation compared to control (Fig. 3B). Control and knock down cells reached the same low level of surface KIT by 90 minutes post stimulation (Fig. S1B). RIN3 silencing was confirmed by immunoblot (Fig. 3C). These results indicated that RIN3 plays a role in setting the rate at which activated KIT is internalized in mast cells.

To verify that accelerated KIT internalization was the result of RIN3 silencing, we restored RIN3 expression by transduction with an adenovirus expression vector. The resulting ectopic RIN3 level in transduced cells was higher than endogenous RIN3 in control cells but whole cell lysate levels of KIT were unchanged (Fig. 3E). Cells with restored RIN3 (Fig. 3E) showed a KIT internalization rate close to that seen in control cells, and lower than that of RIN3 silenced cells (Fig. 3D&F). The proportion of cells that retained surface KIT at 20 minutes post stimulation (peak overlap area, Fig. 3D) was greater in cells with restored RIN3 expression, confirming that RIN3 silencing was responsible for accelerated internalization. Hence, the more rapid KIT internalization rate observed in RIN3 siRNA-transfected cells (Fig. 3A) can most easily be explained by a reduction in RIN3 protein level.

Internalized KIT receptors, like other RTKs, can be recycled back to the cell surface in order to return the cell to an SCF-responsive condition. To examine KIT recycling, LAD2 cells were starved overnight, stimulated for 90 minutes with 5 ng/mL SCF and then placed in medium without SCF. Cells that had been starved of SCF showed high levels of KIT on their surface (Fig. S1B). For cells that were stimulated with SCF and then allowed to incubate in SCF-free medium for 0, 45, or 90 minutes, we observed a gradual recovery of surface KIT. The recovery rate for control and RIN3 silenced cells was indistinguishable. Hence, RIN3 does not appear to directly control the rate of KIT receptor recycling back to the surface post SCF stimulation.

### RIN3 Positively Regulates RAB5 Activation in Mast Cells

**Figure 4:**
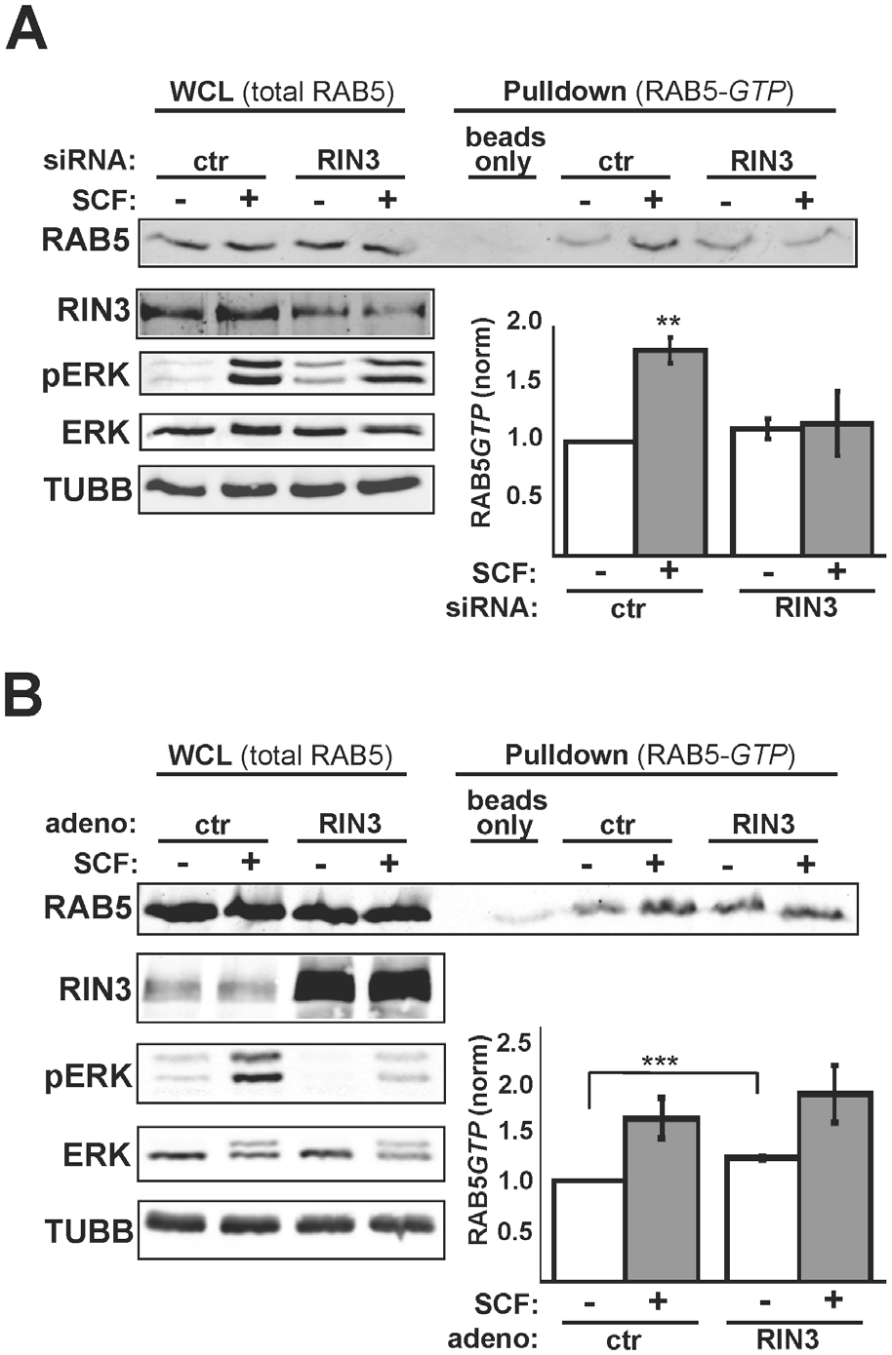
RIN3 promotes RAB5 activation. ( A) LAD2 cells transfected with control or RIN3 siRNA were stimulated with 100 ng/ml SCF for 15 minutes. Cells were lysed and activated RAB5 was pulled down using 4X-ZFYVE-GST on glutathione beads as indicated in Methods. Precipitates from the beads were immunoblotted for RAB5 (RAB5*GTP*). The whole cell lysates were immunoblotted for total RAB5, RIN3, pERK (to show stimulation), ERK, and TUBB (loading control). Graph represents data from three independent experiments, **p<0.01. (B) Cells infected with Ad-GFP or Ad-RIN3 and treated as in (A). Graph represents data from three independent experiments, ***p<1 × 10^−5^.

Because RIN3 is a known GEF for the RAB5 family of small GTPases [19], we examined whether the levels of activated RAB5 correlated with RIN3 expression levels. In order to measure activated endogenous RAB5 we utilized a 4×ZFYVE-GST construct that contains four copies of the 40 amino acid zinc finger domain of the RAB5 effector Rabenosyn that preferentially binds to the active (GTP-bound) conformation of RAB5. There was no significant difference between the basal levels of active RAB5 in control versus RIN3 knock down cells (Fig. 4A). SCF stimulation, which was confirmed by elevated pERK levels, led to higher levels of active RAB5 in control cells (average of 80% increase, p<0.001). In contrast, there was no significant change in the amount of active RAB5 in RIN3 silenced cells (p>0.80) following SCF stimulation. As a result, following SCF treatment there was significantly less active RAB5 in the RIN3 silenced cells than in control cells (p<0.025). Less activated RAB5 in the cell post stimulation would be expected to reduce early endosome fusion. Under these circumstances, internalized KIT may stay in the early endosome for a prolonged period.

We next measured RAB5 activation in RIN3 over-expressing LAD2 cells. As in the previous experiments, SCF stimulation of control cells caused an increase in the level of active RAB5. Even before SCF stimulation, however, RIN3 transduced cells had higher levels of active RAB5 (Fig. 4B, 20% average increase, p<1×10^−5^), presumably due to stimulus-independent GEF activity from over-expressed RIN3. There was no significant difference in the amount of activated RAB5 post stimulation between control and RIN3 over-expressing cells (p>0.2), however, which might indicate a ceiling for RAB5(GTP) levels due to negative regulation by RAB5-directed GAPs.

### RIN3 Over-expression Promotes KIT Down Regulation

**Figure 5:**
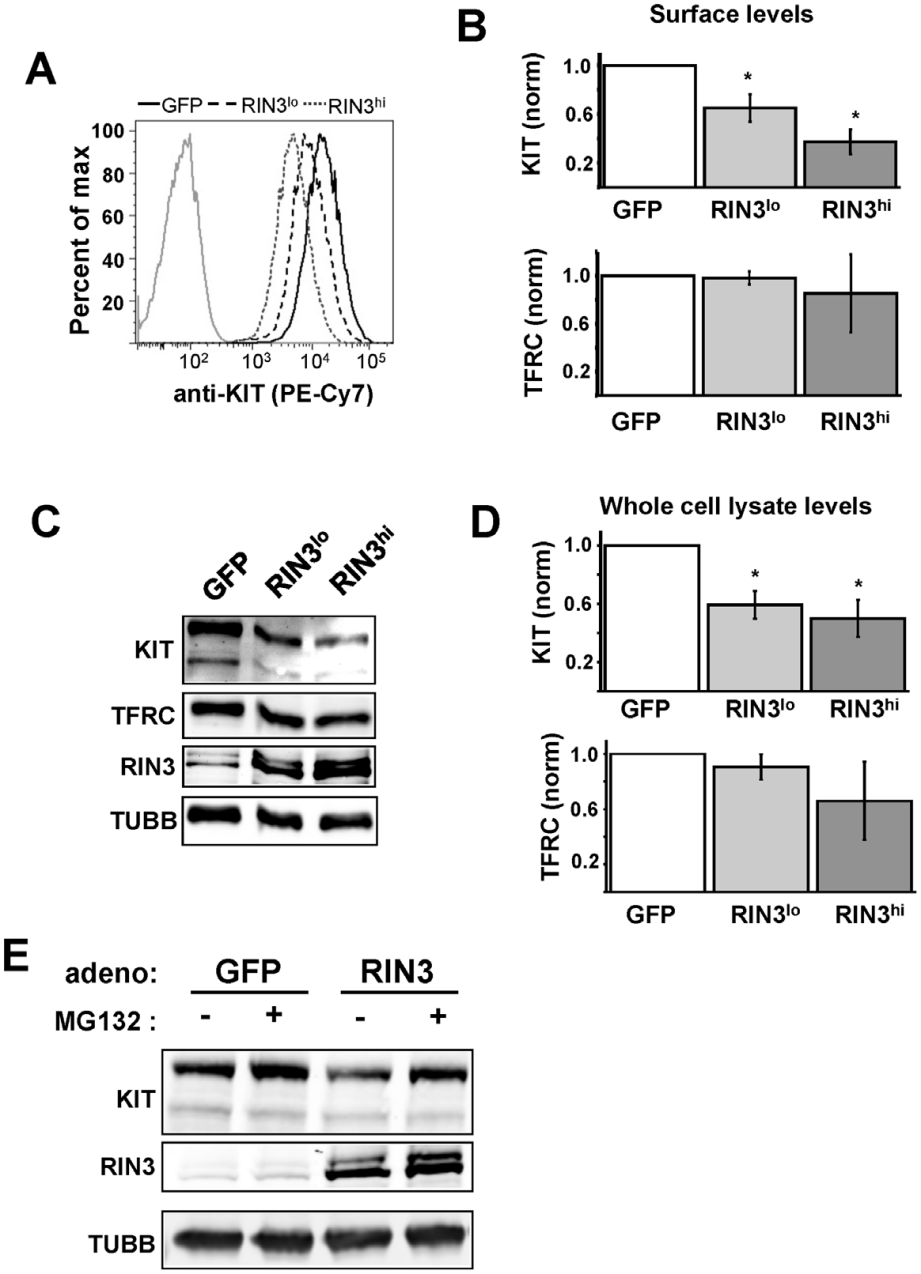
KIT down regulation is enhanced by RIN3 activity. (A) Cells infected with Ad-GFP (black line) or Ad-RIN3 at high (dotted line) and low (dashed line) concentrations were stained with PE/Cy7 labeled anti-KIT and analyzed by flow cytometry for basal surface expression of KIT. Gray line represents unstained control. (B) Graphs showing surface intensity of anti-KIT and anti-TFRC as determined by flow cytometry. Surface expression of GFP transduced cells was set to one. Data are compiled from two independent experiments, *p<0.05. (C) Lysates from cells from A and B were immunoblotted to determine the amount of RIN3 over-expression and basal KIT and TFRC levels. (D) Graphs showing intensity of immunoblot signal for KIT and TFRC compiled from two separate experiments, *p<0.05. (E) The proteasome inhibitor MG132 partially restores KIT expression. Immunoblot for KIT levels in cells incubated for 3 hours with vehicle (ethanol) or 10 µM MG132 after overnight SCF starvation. This immunoblot is representative of two independent experiments; KIT levels increased by 24.5±12.5% for GFP infected cells and 47.5±3.5% for RIN3 infected cells.

In contrast to the accelerated KIT internalization observed in RIN3 silenced mast cells, RIN3 over-expression in the same cells had no effect on the rate of KIT internalization following stimulation with SCF (data not shown). We noted, however, that unstimulated RIN3 over-expressing cells had reduced surface KIT expression compared to control cells. The reduction of surface KIT, as determined by flow cytometry (Fig. 5A and B), was dependent on the relative level of RIN3 over-expression (Fig. 5C). When RIN3 was moderately over-expressed (RIN3^lo^) surface KIT was significantly reduced compared to cells expressing GFP; this reduction was even more pronounced in cells with high RIN3 over-expression (RIN3^hi^) (Fig 5B, p<0.05, p<0.025). As a control, the relative levels of transferrin receptor (TFRC) were measured under the same conditions. High-level over-expression of RIN3 did not significantly alter TFRC surface expression (Fig 5B). In these same cells, we also tested to see if whole cell lysate levels of KIT and TFRC were changed with increasing RIN3 levels. We observed that total cellular KIT decreased markedly as RIN3 levels increased (Fig. 5C&D); however, TFRC levels remain largely unaffected, with a slight decrease at the highest level of RIN3 expression. Previous work [38] has shown that expression of constitutively active RAB5 decreases the surface and whole cell lysate levels of EGFR in HeLa cells. Like EGFR, KIT normally undergoes continuous cycles of internalization with endosome-localized receptors either returned to the cell surface or degraded. This persistent receptor turnover occurs through RAB5 mediated endocytosis, and suggests that RIN3 over-expression, which leads to higher basal levels of activated RAB5, is promoting receptor degradation over recycling. To test this we incubated control or RIN3 over-expressing LAD2 cells with the proteasome inhibitor MG132. Control cells showed a modest increase in KIT levels, while RIN3 over-expression cells showed a more dramatic increase in KIT levels (Fig. 5E). This result suggests that RIN3 over-expression leads to decreased KIT levels due to increased degradation.

### RIN3 Negatively Regulates Cell Migration Toward SCF

**Figure 6:**
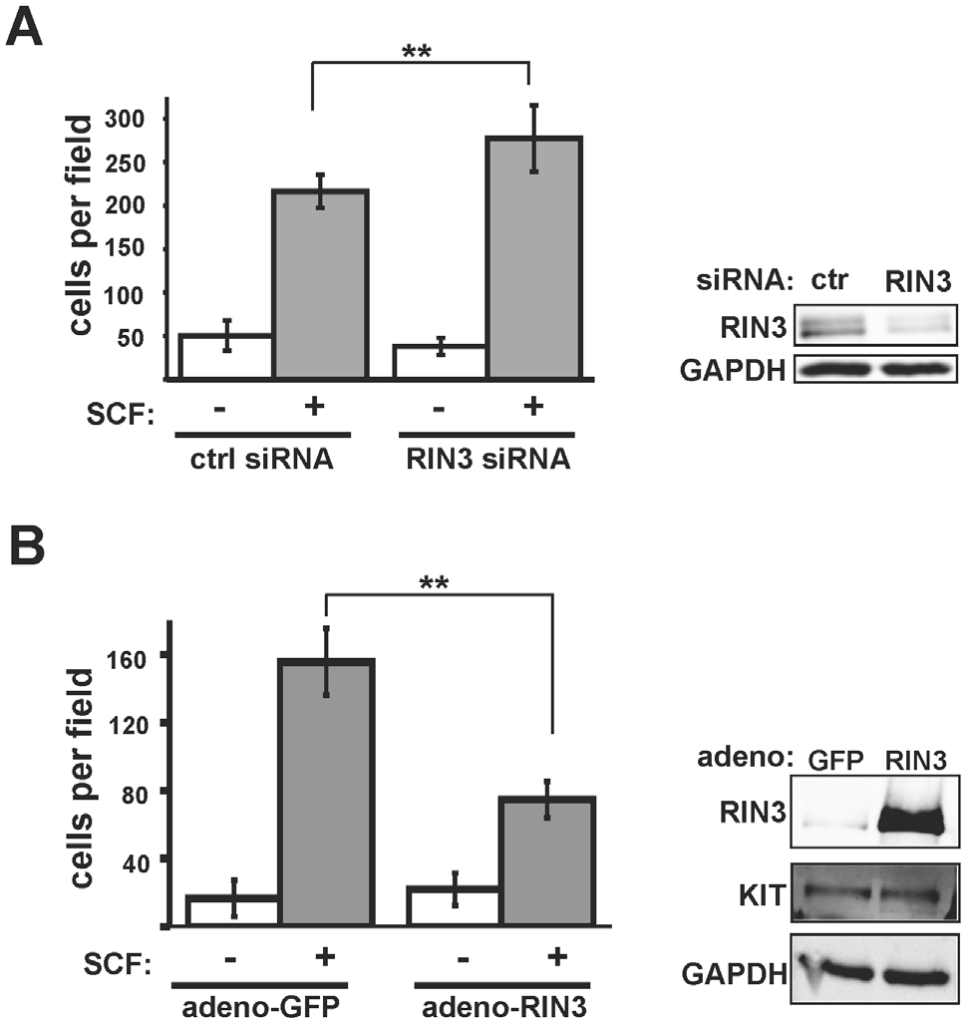
RIN3 inhibits mast cell migration toward SCF. (A) LAD2 cells transfected with control or RIN3 siRNA were allowed to migrate toward SCF. This experiment was performed twice in triplicate, **p<0.01. (B) Cells infected with Ad-GFP or Ad-RIN3 were allowed to migrate toward SCF. Experiment was performed in triplicate, **p<0.01. Immunoblots for RIN3 and TUBB are shown to the right of each graph. Immunoblot quantification (LI-COR) indicated a 26% reduction in KIT (normalized to GAPDH).

Activated mast cells release SCF, the KIT ligand, which recruits more mast cells to sites of allergen infiltration and infection. Because RIN3 silencing caused accelerated KIT internalization, we asked whether RIN3 might also influence the physiological response of mast cells to SCF. To examine this, we measured the ability of mast cells with altered RIN3 expression levels to migrate toward SCF. These experiments were performed using high (100 ng/ml) SCF to elicit a robust migratory response. Control cells showed approximately four-fold more migration toward SCF medium compared to SCF-free medium (Fig. 6A). RIN3 silenced mast cells exhibited a modest but significant increase in migration toward SCF (ctrl siRNA: 217±19 cells; RIN3 siRNA: 277±38 cells, p<0.01), but exhibited no difference from control cells in their rate of migration toward SCF-free medium (Fig. 6A; p>0.15), demonstrating the KIT-dependence of this effect.

To test whether RIN3 functions as a negative regulator of mast cell migration, we established RIN3 over-expressing mast cells by adenovirus transduction. Migration of these cells toward SCF was markedly reduced when compared with mast cells infected with a control adenovirus expressing GFP (Fig. 6B). In these experiments RIN3 over-expression resulted in a two-fold reduction in migration (Ad-GFP: 156±20 cells; Ad-RIN3∶75±11 cells; p<0.01). As noted for RIN3 silenced cells, RIN3 over-expression did not alter the rate of basal cell migration toward SCF-free medium, suggesting that basic cell motility functions were unaffected.

### RIN3 Sensitizes HMC1.1 Mastocytosis Cells to the KIT Inhibitor Imatinib

**Figure 7:**
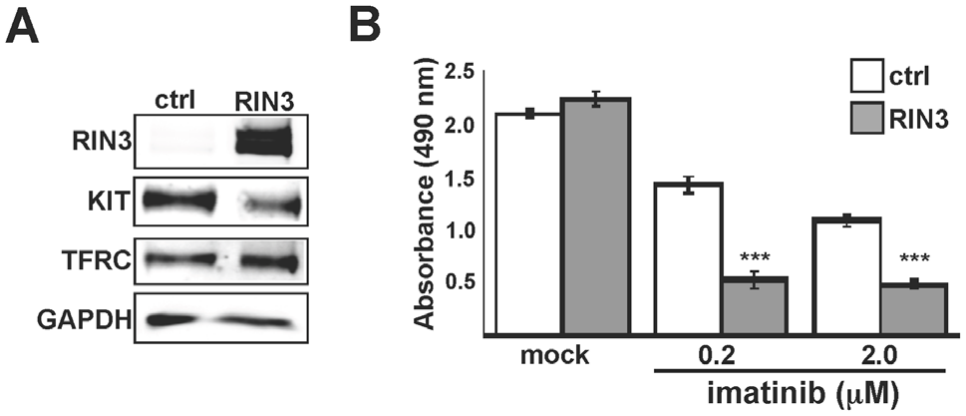
RIN3 over-expression sensitizes mastocytosis cells to imatinib. ( A) HMC1.1 cells infected with empty vector (ctrl) or RIN3 lentivirus were incubated with increasing amounts of Imatinib for 24 hours. (B) The graph represents MTS assay results for cell viability. Assays were performed in quadruplicate, ***p<1 × 10^−5^.

The most common genetic alterations in mastocytosis are mutations that activate KIT and confer a degree of SCF-independent growth. Imatinib, which inhibits KIT and several other tyrosine kinases, provides therapeutic benefits for some mastocytosis patients. We tested whether RIN3 over-expression, which causes KIT down regulation, might increase imatinib sensitivity. HMC1.1 is an established mastocytosis cell line with a KIT^V560G^ mutation. HMC1.1 cells show moderate sensitivity to imatinib with an EC50 of 50–150 nM [39]. HMC1.1 cells were transduced with a RIN3 lentivirus vector to create stable over-expression cells, which showed a reduction in steady state KIT levels (Fig. 7A). Importantly, we observed no effect of RIN3 over-expression on cell proliferation in the absence of drug (Fig. 7B). After a 24-hour incubation in medium containing 0.2 or 2 µM imatinib, control cells showed a drop in viability as determined by MTS assay (Fig. 7B). The HMC1.1 cells over-expressing RIN3 showed an additional, synergistic reduction in cell viability. Hence, by facilitating KIT down regulation, RIN3 sensitizes mastocytosis cells to the therapeutic kinase inhibitor imatinib. RIN3 silencing produced no significant difference in imatinib sensitivity (data not shown), perhaps because the relative drug resistance of these cells is influenced by many factors.

### RIN3 Silencing does not Affect Degranulation

Finally, we examined whether RIN3 is involved in regulating signals from FcεRI, the high affinity IgE receptor on mast cells. FcεRI activation by antigen (Ag)-clustered IgE causes rapid mast cell degranulation with the release of histamine and other inflammation mediators. Degranulation was measured as β-hexosaminidase release following treatment of LAD2 cells with biotinylated IgE and streptavidin (Fig. S2). We observed no significant difference in degranulation timing or intensity when comparing RIN3 silenced cells and control cells (0 ng/ml p>0.66, 1 ng/ml p>0.90, 5 ng/ml p>0.49, 10 ng/ml p>0.51, 100 ng/ml p>0.40). It remains possible that RIN3 influences other events downstream of FcεRI activation, such as migration or secretion, but this determination would require extensive investigation beyond this study.

## Discussion

The limited expression profile of RIN3 is unique among the RIN family proteins that function as RAS effectors and RAB5 activators. Our observation that RIN3 is enriched in, and largely restricted to, mast cells suggests that it evolved to provide a function specific to endocytosis of mast cell receptors. RABGEF1, which includes a RAB5 GEF domain but is otherwise unrelated to RIN proteins, is also found in mast cells ([29] and this work). In contrast, however, the *RABGEF1* gene shows a broad pattern of expression that is more consistent with a basic trafficking function utilized by a wide range of cell types.

**Figure 8:**
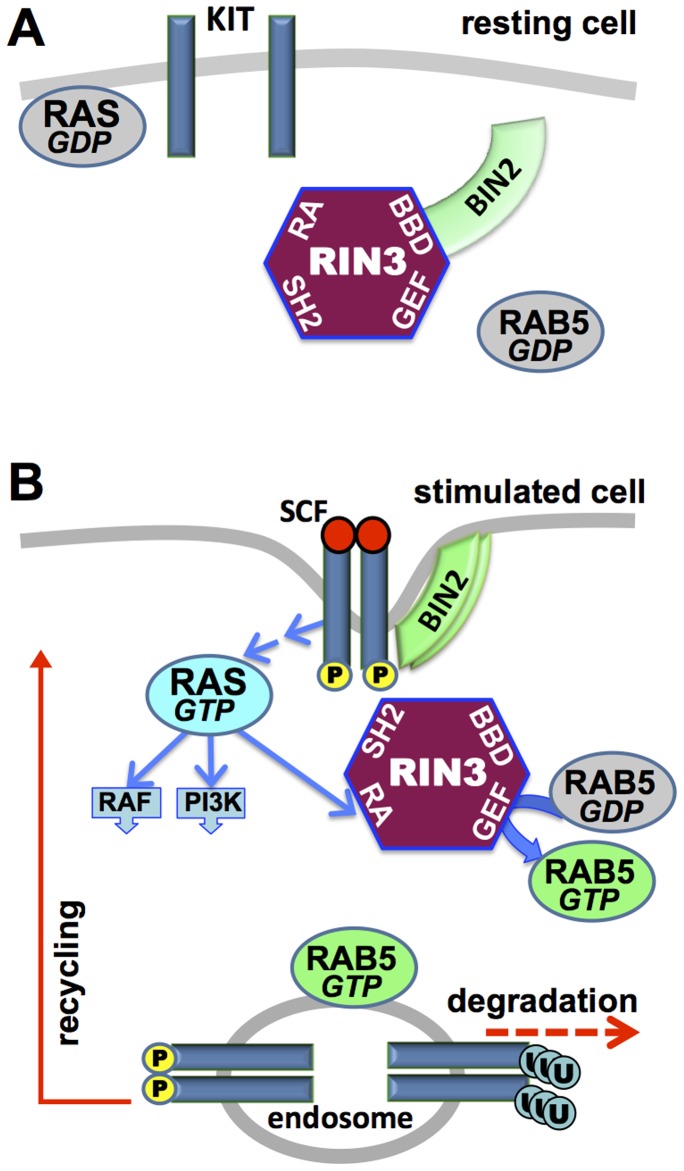
Model of RIN3 function in mast cells. (A) In resting cells BIN2 is bound to RIN3 at a proposed BIN2 Binding Domain (BBD). (B) SCF binding to KIT stimulates RAS. RAS activates RIN3, though a RAS Association (RA) domain, and other effectors (RAF, PI3K). RIN3 releases BIN2, which might then become available for membrane association. The RIN3 GEF domain activates RAB5 to promote early endosome fusion and trafficking. Internalized KIT may continue signaling, be recycled to the plasma membrane, or be targeted for degradation. P = tyrosine phosphorylation; U = ubiquitination; SH2 = Src homology 2 domain (not examined in this work).

The observation that RIN3 silencing accelerates internalization of stimulated KIT was initially surprising. Previous work had reported that mast cells from *Rabgef1* ^−/−^ mice show delayed KIT internalization [29]. This difference highlights the importance of considering GEF activity in the context of the entire protein. In addition to its VPS9 type (RAB5-directed) GEF domain, RABGEF1 has a zinc finger domain that has been implicated in targeting HRAS for ubiquitination [40]. By contrast, the much larger RIN3 protein includes an RA (Ras-Association) domain, SH2 domain and prominent PR (proline-rich) domain. Of particular relevance, we show that RIN3 interacts with BIN2, an amphiphysin related protein with a BAR domain typically used to promote membrane bending during endocytosis and vesicular trafficking. BIN2 was bound to RIN3 in resting cells and the two proteins dissociated soon after KIT stimulation by SCF, but the complex was re-formed after stimulated cells returned to a resting state. We propose a working model (Fig. 8) as a basis for probing signaling events triggered by KIT stimulation and RAS activation. RAS would then recruit and stimulate effector proteins, including RIN3. This enhances the RAB5 GEF activity of RIN3 while also causing the release of BIN2. Although our data do not directly address the role of BIN2 in subsequent events, BAR domain proteins are known to promote plasma membrane bending of the type required during endocytosis. BAR domain proteins can dimerize with other family members and can also form homodimers [41]. Given the low level of BIN1 in LAD2 cells, a BIN2 homodimer might well be operating in this system. Notably, BIN2 expression is restricted to hematopoietic granular cells [35] and mast cells (this work), suggesting an evolved partnership with RIN3.

Highly over-expressed RIN3 led to reduced steady state levels of total and surface resident KIT protein, likely due to elevated levels of endogenous activated (GTP-bound) RAB5. Similar reductions of receptor tyrosine kinases (RTKs) have been seen upon over-expression of related RAB5 GEF domain proteins: RINL over-expression can reduce the basal level of EphA8 [42] and RIN1 over-expression can decrease EGFR protein levels [43]. In unstimulated wild type cells, RTKs such as KIT are typically internalized and recycled back to the plasma membrane. When RAB5 activity is elevated, early endosomes with internalized KIT are more likely to undergo fusion and follow a receptor degradation pathway. This interpretation is supported by the rescue of KIT levels in RIN3 over-expressing cells treated with a proteasome inhibitor. Total KIT levels were unchanged when RIN3 was silenced, but under these conditions we observe very little RAB5 activity pre- or post-SCF stimulation. RIN3 silencing had no affect on mast cell degranulation following activation of the high affinity IgE receptor, FcεRI. This is notable because, like KIT, FcεRI activates RAS and its effector proteins. In addition, *Rabgef1* knockout [30] and Rab5a knock down [44] each enhance IgE-induced degranulation of bone marrow derived mast cells.

RIN3 silenced cells exhibited greater migration toward SCF. These cells internalized KIT more quickly than normal and they have lower levels of active RAB5 following SCF treatment. This might lead to a prolonged stay in the early endosome, with more downstream signaling and a greater chance for KIT recycling back to the plasma membrane while migrating toward SCF. Conversely, RIN3 over-expressing mast cells demonstrated less migration toward SCF. In these cells, decreased levels of surface and total KIT, which correlate with elevated basal levels of active RAB5, would be expected to diminish downstream signaling and biological output. There was no difference in migration toward SCF-free medium when RIN3 was silenced or over-expressed. This implies that changes in migration are due to SCF mediated responses and not due to changes in affinity for fibronectin. The ability to migrate toward SCF is fundamental to mast cell function, suggesting that a deeper understanding of RIN3 signaling may provide new avenues for intervention in mast cell pathophysiology.

KIT gain-of-function mutations are common in mastocytosis and a limited number of other hyperproliferative disorders. Imatinib is an effective tyrosine kinase inhibitor for KIT, as well as ABL and PDGF receptor [45]. This drug has been used with considerable success in the treatment of *BCR-ABL1* positive chronic myeloid leukemias [46]. Imatinib has also shown some effectiveness in the treatment gastrointestinal tumors with mutant KIT [47]. In addition, imatinib has been shown to prevent and lessen the symptoms of rheumatoid arthritis [48], an inflammatory joint disease with mast cell involvement [49]. Previous studies have established that reduced KIT expression, in conjunction with tyrosine kinase inhibitors, can decrease proliferation and increase apoptosis in the mastocytosis cell line HMC1.1, which carries the KIT^V560G^ activating mutation [50]. We demonstrate here that RIN3 over-expression markedly sensitizes HMC1.1 cells to imatinib treatment. This may open new avenues for synergistically enhancing the potency of KIT tyrosine kinase inhibitors. Such combination therapies would be applicable to malignancies characterized by KIT gain-of-function mutations, as well as chronic inflammation pathologies with mast cell involvement.

## Materials and Methods

### Antibodies

To make human RIN3 antibodies, the full length protein was first expressed as a 6 × His fusion from a baculovirus expression vector in Sf9 cells. Metal affinity chromatography (Ni-NTA beads, Qiagen) purified protein was used to generate rabbit polyclonal antibodies (21^st^ Century Biochemicals) that were used for immunoblot staining at a 1/5,000 dilution, for immunoprecipitation at a 1/250 dilution and for immunohistochemistry at 1/5,000.

Other antibodies, their sources and the dilutions used for immunoblot probing were: rabbit anti-RIN1 [51] 1/1,000; rabbit anti-RAB5 (Abcam ab18211) 1/250; rabbit anti-RAS (Novus Bio EP1125Y) 1/5000; rabbit anti-KIT (Cell Signaling 3392) 1/500; mouse anti-KIT (Abcam Ab81) 1/500; mouse anti-α tubulin (Sigma) 1/3000; mouse anti-KIT conjugated to PE/Cy7 (Biolegend 104D2) 1/100; rabbit anti-GAPDH (Abcam) 1/3000; mouse anti-pTyr clone 4G10 (Millipore) 1/500; rabbit anti-RABGEF1 (Sigma) 1/1,000; rabbit anti-BIN2 [35] 1/3000; mouse anti-transferrin receptor (Invitrogen) 1/500; anti MCT (DakyCytomation) 1/2000.

### Cell Culture

The human cell lines used in this work (LAD2, LUVA, HMC1.1 and HMC1.2) were previously established, have been described in the published work cited below and were obtained with institutional approval. The LAD2 cell line [37] was a generous gift from A. Kirshenbaum (NIH, Bethesda, MD). They were maintained in StemPro with 100 ng/ml SCF at less than 500,000 cells/ml. The HMC1.1 and HMC1.2 cell lines were generous gifts from Dr. J.H. Butterfield (Mayo Clinic, Rochester, MN) [52] maintained in Iscove’s DMEM with 10% FBS, 1% glutamax, and 0.01% mono-thioglycerol. The LUVA cell line was a generous gift from J. Steinke (Asthma and Allergic Diseases Center, University of Virginia) [53] maintained in StemPro without SCF. Parental HMC cells were obtained from Dr. Chad Oh (Department of Pediatrics, Harbor-UCLA Medical Center).

### Degranulation Assay

Degranulation was determined by amount of β-hexosaminidase released as previously described [37]. LAD2 cells were primed with 100 ng/mL biotinylated IgE (Abbiotec) in HEPES buffer (10 mM HEPES, 137 mM NaCl, 2.7 mM KCl, 0.4 mM Na2HPO_4_, 5.6 mM glucose, 1.8 nM CaCl_2_, 1.3 nM MgSO_4_ pH 7.4)+0.4% BSA for 1 hour at 37°C. Cells were washed three times and resuspended in HEPES + BSA at 20,000 cells per well. Indicated amount of streptavidin (Sigma) was added to each well; each concentration was done in triplicate. After 30 minutes incubation, cells were pelleted and lysed in 1% Triton X-100 in PBS. Supernatants were aliquoted into a 96 well plate; β-hexosaminidase activity for both the supernatants and the lysates were determined by incubation with p-nitrophenyl N-acetyl-β-D-glucosamide. Percent release was calculated as amount in supernatant/(supernatant + lysate).

### Immunoprecipitation and Immunoblotting

For immunoblotting, proteins were transferred to ECL membranes and blocked with 5% milk in PBS. Primary antibodies were incubated overnight at 4°C in 5% milk in PBST (PBS+0.1% Tween). This was followed by three washes in PBST and one hour incubation with secondary antibodies at room temperature. This was true for all antibodies with the exception of 4G10 antibody, which was used according to Millipore’s recommendations. Membranes were developed using the ECL plus immunoblotting reagent (VWR). Immunoblots exposed on film were quantified using ImageJ software. All other immunoblots were quantified using a Li-Cor Odyssey scanner and software.

For immunoprecipitation experiments, LAD2 cells were starved of SCF in complete StemPro media overnight, then stimulated with SCF for indicated time points and concentrations of growth factor. Cells were centrifuged, washed once in PBS, and lysed in NP-40 buffer (150 mM NaCl, 50 mM Tris pH 8.0, 1% glycerol, 1% NP-40) on ice for 15 minutes. Lysates were spun at 16000 × g and the pellet was discarded. To enrich for RIN3 and its binding partners, RIN3 antibody was added to cell lysate with protein A and protein G beads and incubated at 4°C with rotation for three hours to overnight. Immunoprecipitated samples were gently spun down at 800×g and the pelleted beads were washed twice in lysis buffer. Beads were boiled in 5× SDS loading buffer to release proteins from beads. Samples were then analyzed by SDS PAGE and immunoblotting.

### Expression Constructs, Lentiviruses and Adenoviruses

RIN3 cDNA (Open Biosystems) was PCR amplified and cloned into pBluescript KS to create pKS-RIN3. A FLAG tag was fused onto the C-terminus of the protein to create RIN3-FLAG. This was subsequently inserted into an M4 vector that was used for lentiviral production as previously described [36].

A 6 × Histidine tag was added to RIN3 using the following oligonucleotides 5′- TCGACCACCACCATCACCATCACCATCACTAACTAGT and 5′- GTAGACTAGTTAGTGATGGTGATGGTGATGGTGGTGG. RIN3-His was then cloned into the donor vector pFastBac (Invitrogen) in order to produce baculovirus for Sf9 cell infections following established procedures.

Adenoviral vector construction was performed by homologous recombination in *E. coli*, as previously described [54]. The Ad5/F35 viral vector contains a 2,115-bp-long chimeric fiber gene consisting of the Ad5 tail and the Ad35 shaft and knob domains [55]. RIN3-FLAG was cloned into the pShuttle-CMV plasmid and transformed into recombinogenic *E. coli* strain BJ5183 that had already been transformed with a plasmid containing the Ad5/F35 vector genome. Full-length recombinant adenoviral vectors genomic plasmids were amplified after re-transformation into Stabl3 cells, and transfected into Ad293 cells to generate an initial virus stock. The virus was then propagated in progressively larger scale cell cultures, and after the final amplification step, the virus was purified using an AdEasy Purification kit (Clontech). A TCID50 assay was performed to determine the titer (IU/ml) of the working virus stock. Low and high RIN3 over-expression cell lines were created by adjusting the multiplicity of infection for adenovirus and LAD2 cells (range from 2 to 20 infectious units per cell).

### Flow Cytometry

To measure internalization, LAD2 cells were starved of SCF overnight and stimulated with 5 ng/ml SCF at 37°C for times indicated. Cells were spun down, washed once in cold PBS. For the KIT recovery assay LAD2 cells were starved of SCF overnight and stimulated with 5 ng/ml SCF for 90 minutes at 37°C. Cells were spun down, washed once in room temperature PBS and resuspended in fresh StemPro without SCF. They were incubated at 30°C for times indicated then washed in cold PBS. Staining was performed the same for both assays: cells were resuspended in PBS+0.1% BSA with PE/CY7-anti-KIT (Biolegend 104D2) diluted 1/100 or Alexa Fluor 647-anti-CD71 (Santa Cruz) diluted 1/50 and incubated for 45 minutes at 4°C. After washing once in PBS+0.1% BSA, they were passed through a 2 µm filter, and run on FACS (BD FSR II) using BD FACS Diva software. Results were analyzed using FlowJo. FACS graphs were gated on the viable cell population.

### RIN3 Knockdown

Knock down experiments were conducted using the ON-TARGETplus system from Dharmacon. The non-targeting siRNA (ON-TARGETplus Non-targeting siRNA #3) and siRNA targeted to RIN3 (GCAGCAUGUUCCACGCUUU) were transfected with Dharmafect 1 according to manufacturer’s directions. The final concentration of RNA in each culture was 3 nM; LAD2 cells were at a concentration of 1.25 × 10^5^ cells/ml. To restore RIN3 expression, the Ad-RIN3 adenovirus was added 24 hours post siRNA transfection.

### Immunohistochemistry

Immunohistochemical analysis was performed on formalin-fixed, paraffin-embedded sections from mastocytosis patients (UCLA Translational Pathology). Sections were de-waxed at 60°C and rehydrated with Safeclear and ethanol, then incubated in 0.5% trypsin at 37°C for 20 minutes for antigen retrieval. Endogenous peroxidase activity was blocked with 3% H_2_O_2_ for 15 minutes. Samples were blocked in 5% goat serum for 30 minutes before being incubated overnight with anti-RIN3 or anti-MCT. Slides were washed three times in PBS and incubated with biotinylated goat anti-rabbit secondary for 40 minutes. Staining was performed using avidin-biotin complex (ABC, Vectastain) and detected using 3,diaminobezidine (DAB, Vectastain). Samples were counterstained with hematoxylin and neutralized with ammonia hydroxide. Slides were then dehydrated and coverslips were attached with Permamount.

### Migration Assay

Migration assays were performed using a modified Boyden Chamber (8.0 um pore size). The bottom of the chamber was coated overnight with 800 ng/mL fibronectin. LAD2 cells were starved of SCF overnight and resuspended at 5 × 10^5^ cells/mL in fresh media without SCF. Chambers were place above media with or without 100 ng/mL SCF and allowed to migrate for 1 hour at 37°C. Chambers were then washed in PBS, fixed with 4% PFA for 15 minutes, and stained with 0.1% crystal violet in 10% ethanol for 20 minutes. Cells were wiped from the top chamber that was then washed in PBS to remove excess stain. Cells were counted by light microscopy.

### Activated RAB5 Pull Down

BL21 cells were transformed with pGEX 4×ZFYVE-GST, a tagged RAB5 effector domain (Balaji and Colicelli, unpublished). After induction for 3 hours at 37°C, cells were lysed by sonication in buffer containing 20 mM Tris, 250 mM NaCl, 10% glycerol, and 0.01% Triton. Glutathione sepharose was added to lysate and incubated for 1 hour. Beads were centrifuged and resuspended in NP-40 lysis buffer. Cells were prepared with SCF starvation overnight in then 0 or 5 minutes of 100 ng/mL SCF treatment at 37°C. Cells were then spun down, washed once in cold PBS and lysed in NP-40 buffer with the addition of 1 mM DTT and 10 mM MgCl_2_. Glutathione beads preloaded with effector were added to each lysate and allowed to bind for one hour followed by immunoblot detection of total RAB5.

### MTS Assays

HMC1.1 cells were plated at 50,000 cells per 100 µl in each well of 96 well plate. Imatinib was added at 0, 0.2, and 2 µM to each well. Cells were incubated at 37°C, 5% CO_2_ for 24 hours. Twenty microliters of MTS AQ reagent (Promega) was added to each well, mixed, and incubated for 3 hours at 37°C, 5% CO_2_. Absorbance at 490 nm was read on (Victor^3^, 1420 Multilabel Counter, PerkinElmer) and normalized to media only control.

## Supporting Information

Figure S1 **RIN3 silencing does not affect KIT surface recovery.** (A) Representative blot comparing levels of RIN3 in mock, ctrl siRNA, and RIN3 siRNA transfected cells. Transfection with siRNA did not affect wild type levels of RIN3. (B) Cells were transfected with control (blue) or RIN3 siRNA (red). Surface expression of KIT was measured by flow cytometry before stimulation (top left), after stimulation with 5 ng/ml SCF (top right) and at two time points of recovery in SCF free media (bottom panels). Gray line represents unstained control. Immunoblot indicates level of RIN3 in lysates.
(PDF)

Figure S2 **RIN3 silencing does not affect degranulation.** The amount of granule release was measured in cells with control (white bars) or knock down levels of RIN3 (gray bars). All cells were primed with biotinylated IgE and then incubated with indicated concentrations of streptavidin (antigen). Percent granule release was calculated as β-hexosaminidase activity: supernatant/(supernatant + lysate). Graph is compilation of two independent experiments performed in triplicate.
(PDF)

## References

[1] Gilfillan AM, Beaven MA (2011) Regulation of mast cell responses in health and disease. Crit Rev Immunol 31: 475–529. 22321108 10.1615/critrevimmunol.v31.i6.30 PMC3395887

[2] Amin K (2012) The role of mast cells in allergic inflammation. Respir Med 106: 9–14. 22112783 10.1016/j.rmed.2011.09.007

[3] Ronnstrand L (2004) Signal transduction via the stem cell factor receptor/c-Kit. Cell Mol Life Sci 61: 2535–2548. 15526160 10.1007/s00018-004-4189-6 PMC11924424

[4] Roskoski R Jr (2005) Structure and regulation of Kit protein-tyrosine kinase–the stem cell factor receptor. Biochem Biophys Res Commun 338: 1307–1315. 16226710 10.1016/j.bbrc.2005.09.150

[5] Roskoski R Jr (2005) Signaling by Kit protein-tyrosine kinase–the stem cell factor receptor. Biochem Biophys Res Commun 337: 1–13. 16129412 10.1016/j.bbrc.2005.08.055

[6] Campbell E, Hogaboam C, Lincoln P, Lukacs NW (1999) Stem cell factor-induced airway hyperreactivity in allergic and normal mice. Am J Pathol 154: 1259–1265. 10233863 10.1016/S0002-9440(10)65377-1 PMC1866576

[7] Ashman LK (1999) The biology of stem cell factor and its receptor C-kit. Int J Biochem Cell Biol 31: 1037–1051. 10582338 10.1016/s1357-2725(99)00076-x

[8] Boissan M, Feger F, Guillosson JJ, Arock M (2000) c-Kit and c-kit mutations in mastocytosis and other hematological diseases. J Leukoc Biol 67: 135–148. 10670573 10.1002/jlb.67.2.135

[9] Metcalfe DD (2008) Mast cells and mastocytosis. Blood 112: 946–956. 18684881 10.1182/blood-2007-11-078097 PMC2515131

[10] Qualmann B, Koch D, Kessels MM (2011) Let’s go bananas: revisiting the endocytic BAR code. Embo J 30: 3501–3515. 21878992 10.1038/emboj.2011.266 PMC3181480

[11] Rao Y, Haucke V (2011) Membrane shaping by the Bin/amphiphysin/Rvs (BAR) domain protein superfamily. Cell Mol Life Sci 68: 3983–3993. 21769645 10.1007/s00018-011-0768-5 PMC11114942

[12] Pant S, Sharma M, Patel K, Caplan S, Carr CM, et al (2009) AMPH-1/Amphiphysin/Bin1 functions with RME-1/Ehd1 in endocytic recycling. Nat Cell Biol 11: 1399–1410. 19915558 10.1038/ncb1986 PMC2788952

[13] Barbieri MA, Roberts RL, Mukhopadhyay A, Stahl PD (1996) Rab5 regulates the dynamics of early endosome fusion. Biocell 20: 331–338. 9031602

[14] Fukuda M (2008) Regulation of secretory vesicle traffic by Rab small GTPases. Cell Mol Life Sci 65: 2801–2813. 18726178 10.1007/s00018-008-8351-4 PMC11131888

[15] Grosshans BL, Ortiz D, Novick P (2006) Rabs and their effectors: achieving specificity in membrane traffic. Proc Natl Acad Sci U S A 103: 11821–11827. 16882731 10.1073/pnas.0601617103 PMC1567661

[16] Somsel Rodman J, Wandinger-Ness A (2000) Rab GTPases coordinate endocytosis. J Cell Sci 113 Pt 2: 183–192. 10.1242/jcs.113.2.183 10633070

[17] Clague MJ, Urbe S (2001) The interface of receptor trafficking and signalling. J Cell Sci 114: 3075–3081. 11590234 10.1242/jcs.114.17.3075

[18] Navaroli DM, Bellve KD, Standley C, Lifshitz LM, Cardia J, et al (2012) Rabenosyn-5 defines the fate of the transferrin receptor following clathrin-mediated endocytosis. Proc Natl Acad Sci U S A 109: E471–480. 22308388 10.1073/pnas.1115495109 PMC3286945

[19] Kajiho H, Saito K, Tsujita K, Kontani K, Araki Y, et al (2003) RIN3: a novel Rab5 GEF interacting with amphiphysin II involved in the early endocytic pathway. J Cell Sci 116: 4159–4168. 12972505 10.1242/jcs.00718

[20] Hu H, Bliss JM, Wang Y, Colicelli J (2005) RIN1 is an ABL tyrosine kinase activator and a regulator of epithelial-cell adhesion and migration. Curr Biol 15: 815–823. 15886098 10.1016/j.cub.2005.03.049

[21] Kong C, Su X, Chen PI, Stahl PD (2007) RIN1 interacts with signal transducing adaptor molecule (STAM) and mediates epidermal growth factor receptor trafficking and degradation. J Biol Chem 282: 15294–15301. 17403676 10.1074/jbc.M611538200

[22] Tomshine JC, Severson SR, Wigle DA, Sun Z, Beleford DA, et al (2009) Cell proliferation and epidermal growth factor signaling in non-small cell lung adenocarcinoma cell lines are dependent on Rin1. J Biol Chem 284: 26331–26339. 19570984 10.1074/jbc.M109.033514 PMC2785321

[23] Barbieri MA, Kong C, Chen PI, Horazdovsky BF, Stahl PD (2003) The SRC homology 2 domain of Rin1 mediates its binding to the epidermal growth factor receptor and regulates receptor endocytosis. J Biol Chem 278: 32027–32036. 12783862 10.1074/jbc.M304324200

[24] Hu H, Milstein M, Bliss JM, Thai M, Malhotra G, et al (2008) Integration of TGFbeta and RAS Signaling Silences a RAB5 GEF and Enhances Growth Factor-DIrected Cell Migration. Mol Cell Biol 28: 1573–1583. 18160707 10.1128/MCB.01087-07 PMC2258770

[25] Hunker CM, Galvis A, Veisaga ML, Barbieri MA (2006) Rin1 is a negative regulator of the IL3 receptor signal transduction pathways. Anticancer Res 26: 905–916. 16619486

[26] Hunker CM, Giambini H, Galvis A, Hall J, Kruk I, et al (2006) Rin1 regulates insulin receptor signal transduction pathways. Exp Cell Res 312: 1106–1118. 16457816 10.1016/j.yexcr.2005.12.021

[27] Deininger K, Eder M, Kramer ER, Zieglgansberger W, Dodt HU, et al (2008) The Rab5 guanylate exchange factor Rin1 regulates endocytosis of the EphA4 receptor in mature excitatory neurons. Proc Natl Acad Sci U S A 105: 12539–12544. 18723684 10.1073/pnas.0801174105 PMC2527947

[28] Tall GG, Barbieri MA, Stahl PD, Horazdovsky BF (2001) Ras-activated endocytosis is mediated by the Rab5 guanine nucleotide exchange activity of RIN1. Dev Cell 1: 73–82. 11703925 10.1016/s1534-5807(01)00008-9

[29] Kalesnikoff J, Rios EJ, Chen CC, Nakae S, Zabel BA, et al (2006) RabGEF1 regulates stem cell factor/c-Kit-mediated signaling events and biological responses in mast cells. Proc Natl Acad Sci U S A 103: 2659–2664. 16533754 10.1073/pnas.0511191103 PMC1413845

[30] Kalesnikoff J, Rios EJ, Chen CC, Alejandro Barbieri M, Tsai M, et al (2007) Roles of RabGEF1/Rabex-5 domains in regulating Fc epsilon RI surface expression and Fc epsilon RI-dependent responses in mast cells. Blood 109: 5308–5317. 17341663 10.1182/blood-2007-01-067363 PMC1890836

[31] Zhu H, Liang Z, Li G (2009) Rabex-5 is a Rab22 effector and mediates a Rab22-Rab5 signaling cascade in endocytosis. Mol Biol Cell 20: 4720–4729. 19759177 10.1091/mbc.E09-06-0453 PMC2777102

[32] Horiuchi H, Lippe R, McBride HM, Rubino M, Woodman P, et al (1997) A novel Rab5 GDP/GTP exchange factor complexed to Rabaptin-5 links nucleotide exchange to effector recruitment and function. Cell 90: 1149–1159. 9323142 10.1016/s0092-8674(00)80380-3

[33] Lee E, Marcucci M, Daniell L, Pypaert M, Weisz OA, et al (2002) Amphiphysin 2 (Bin1) and T-tubule biogenesis in muscle. Science 297: 1193–1196. 12183633 10.1126/science.1071362

[34] Leprince C, Romero F, Cussac D, Vayssiere B, Berger R, et al (1997) A new member of the amphiphysin family connecting endocytosis and signal transduction pathways. J Biol Chem 272: 15101–15105. 9182529 10.1074/jbc.272.24.15101

[35] Ge K, Prendergast GC (2000) Bin2, a functionally nonredundant member of the BAR adaptor gene family. Genomics 67: 210–220. 10903846 10.1006/geno.2000.6216

[36] Hu H, Milstein M, Bliss JM, Thai M, Malhotra G, et al (2008) Integration of transforming growth factor beta and RAS signaling silences a RAB5 guanine nucleotide exchange factor and enhances growth factor-directed cell migration. Mol Cell Biol 28: 1573–1583. 18160707 10.1128/MCB.01087-07 PMC2258770

[37] Kirshenbaum AS, Akin C, Wu Y, Rottem M, Goff JP, et al (2003) Characterization of novel stem cell factor responsive human mast cell lines LAD 1 and 2 established from a patient with mast cell sarcoma/leukemia; activation following aggregation of FcepsilonRI or FcgammaRI. Leuk Res 27: 677–682. 12801524 10.1016/s0145-2126(02)00343-0

[38] Dinneen JL, Ceresa BP (2004) Continual expression of Rab5(Q79L) causes a ligand-independent EGFR internalization and diminishes EGFR activity. Traffic 5: 606–615. 15260830 10.1111/j.1398-9219.2004.00204.x

[39] Aichberger KJ, Mayerhofer M, Gleixner KV, Krauth MT, Gruze A, et al (2007) Identification of MCL1 as a novel target in neoplastic mast cells in systemic mastocytosis: inhibition of mast cell survival by MCL1 antisense oligonucleotides and synergism with PKC412. Blood 109: 3031–3041. 17110460 10.1182/blood-2006-07-032714

[40] Xu L, Lubkov V, Taylor LJ, Bar-Sagi D (2010) Feedback regulation of Ras signaling by Rabex-5-mediated ubiquitination. Curr Biol 20: 1372–1377. 20655225 10.1016/j.cub.2010.06.051 PMC3436604

[41] Prendergast GC, Muller AJ, Ramalingam A, Chang MY (2009) BAR the door: cancer suppression by amphiphysin-like genes. Biochim Biophys Acta 1795: 25–36. 18930786 10.1016/j.bbcan.2008.09.001 PMC2874822

[42] Kajiho H, Fukushima S, Kontani K, Katada T (2012) RINL, guanine nucleotide exchange factor Rab5-subfamily, is involved in the EphA8-degradation pathway with odin. PLoS One 7: e30575. 22291991 10.1371/journal.pone.0030575 PMC3264577

[43] Balaji K, Mooser CK, Janson CM, Bliss JM, Hojjat H, et al.. (2012) RIN1 Orchestrates the Activation of RAB5 GTPases and ABL Tyrosine Kinases to Determine EGFR Fate. Journal of Cell Science (in press). 10.1242/jcs.113688 PMC3575715 22976291

[44] Kageyama-Yahara N, Suehiro Y, Yamamoto T, Kadowaki M (2011) Rab5a regulates surface expression of FcepsilonRI and functional activation in mast cells. Biol Pharm Bull 34: 760–763. 21532169 10.1248/bpb.34.760

[45] Capdeville R, Buchdunger E, Zimmermann J, Matter A (2002) Glivec (STI571, imatinib), a rationally developed, targeted anticancer drug. Nat Rev Drug Discov 1: 493–502. 12120256 10.1038/nrd839

[46] Druker BJ, Tamura S, Buchdunger E, Ohno S, Segal GM, et al (1996) Effects of a selective inhibitor of the Abl tyrosine kinase on the growth of Bcr-Abl positive cells. Nat Med 2: 561–566. 8616716 10.1038/nm0596-561

[47] Joensuu H, Roberts PJ, Sarlomo-Rikala M, Andersson LC, Tervahartiala P, et al (2001) Effect of the tyrosine kinase inhibitor STI571 in a patient with a metastatic gastrointestinal stromal tumor. N Engl J Med 344: 1052–1056. 11287975 10.1056/NEJM200104053441404

[48] Miyachi K, Ihara A, Hankins RW, Murai R, Maehiro S, et al (2003) Efficacy of imatinib mesylate (STI571) treatment for a patient with rheumatoid arthritis developing chronic myelogenous leukemia. Clin Rheumatol 22: 329–332. 14576993 10.1007/s10067-003-0716-3

[49] Nigrovic PA, Lee DM (2005) Mast cells in inflammatory arthritis. Arthritis Res Ther 7: 1–11. 15642148 10.1186/ar1446 PMC1064877

[50] Jin Y, Lu Z, Cao K, Zhu Y, Chen Q, et al (2010) The antitumor activity of homoharringtonine against human mast cells harboring the KIT D816V mutation. Mol Cancer Ther 9: 211–223. 20053766 10.1158/1535-7163.MCT-09-0468

[51] Han L, Wong D, Dhaka A, Afar D, White M, et al (1997) Protein binding and signaling properties of RIN1 suggest a unique effector function. Proc Natl Acad Sci U S A 94: 4954–4959. 9144171 10.1073/pnas.94.10.4954 PMC24612

[52] Butterfield JH, Weiler D, Dewald G, Gleich GJ (1988) Establishment of an immature mast cell line from a patient with mast cell leukemia. Leuk Res 12: 345–355. 3131594 10.1016/0145-2126(88)90050-1

[53] Laidlaw TM, Steinke JW, Tinana AM, Feng C, Xing W, et al.. (2011) Characterization of a novel human mast cell line that responds to stem cell factor and expresses functional FcepsilonRI. J Allergy Clin Immunol 127: 815–822 e811–815. 10.1016/j.jaci.2010.12.1101 PMC3052637 21281958

[54] He TC, Zhou S, da Costa LT, Yu J, Kinzler KW, et al (1998) A simplified system for generating recombinant adenoviruses. Proc Natl Acad Sci U S A 95: 2509–2514. 9482916 10.1073/pnas.95.5.2509 PMC19394

[55] Shayakhmetov DM, Papayannopoulou T, Stamatoyannopoulos G, Lieber A (2000) Efficient gene transfer into human CD34(+) cells by a retargeted adenovirus vector. J Virol 74: 2567–2583. 10684271 10.1128/jvi.74.6.2567-2583.2000 PMC111745

