# Supplementary material for: RIN3 Is a Negative Regulator of Mast Cell Responses to SCF
Source: PLoS One. 2012 Nov 20;7(11):e49615. doi: 10.1371/journal.pone.0049615 (PMC3502454; doi:10.1371/journal.pone.0049615)
Supplement: Figure S1 — RIN3 silencing does not affect KIT surface recovery. (A) Representative blot comparing levels of RIN3 in mock, ctrl siRNA, and RIN3 siRNA transfected cells. Transfection with siRNA did not affect wild type levels of RIN3. (B) Cells were transfected with control (blue) or RIN3 siRNA (red). Surface expression of KIT was measured by flow cytometry before stimulation (top left), after stimulation with 5 ng/ml SCF (top right) and at two time points of recovery in SCF free media (bottom panels). Gray line represents unstained control. Immunoblot indicates level of RIN3 in lysates. (PDF) [file pone.0049615.s001.pdf]

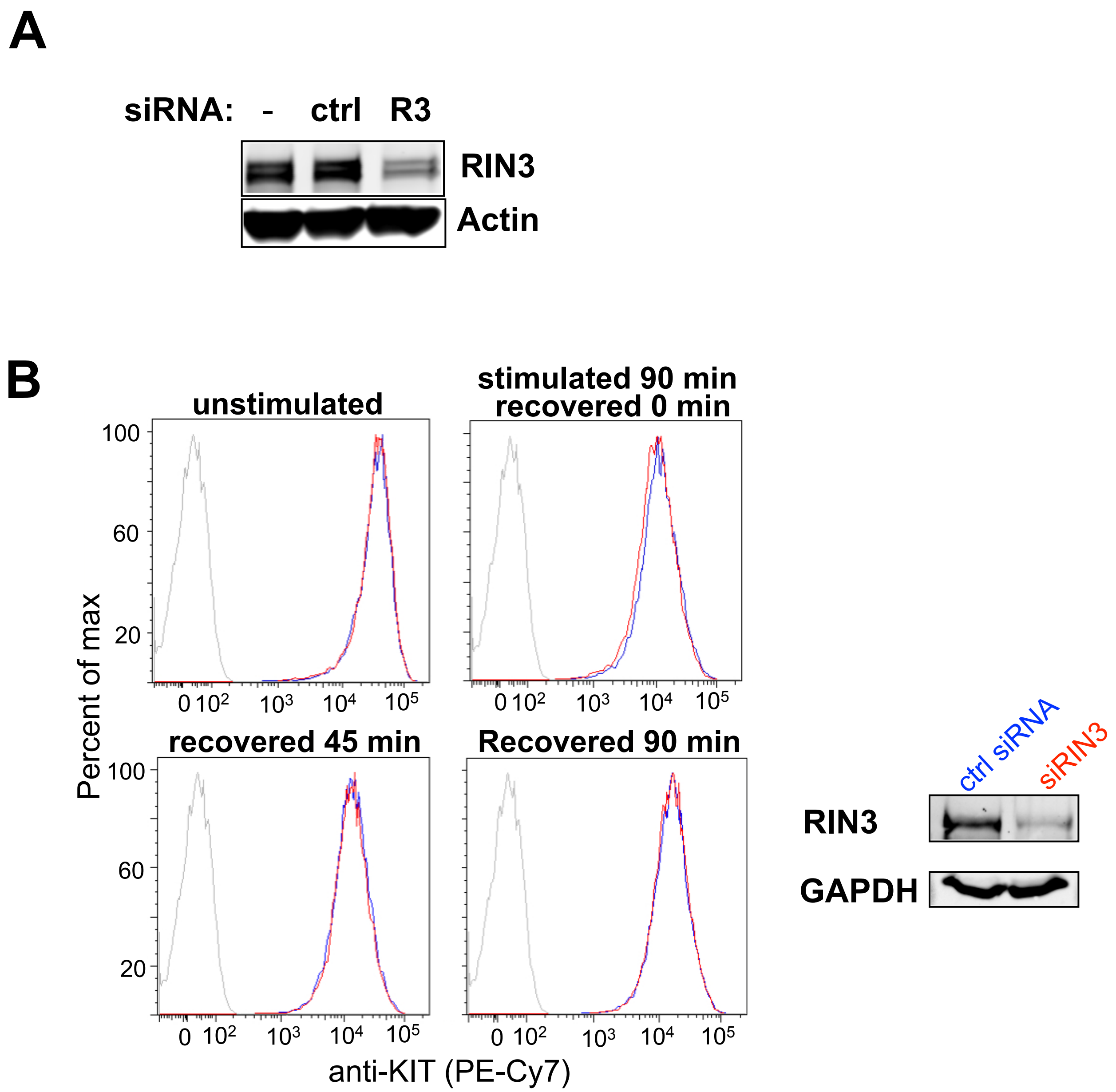

Figure S1. *RIN3 silencing does not affect KIT surface recovery.*
A. Representative blot comparing levels of RIN3 in mock, ctrl siRNA, and RIN3 siRNA transfected cells. Transfection with siRNA did not affect wild type levels of RIN3. **B.** Cells were transfected with control (blue) or RIN3 siRNA (red). Surface expression of KIT was measured by flow cytometry before stimulation (top left), after stimulation with 5 ng/ml SCF (top right) and at two time points of recovery in SCF free media (bottom panels). Gray line represents unstained control. Immunoblot indicates level of RIN3 in lysates.
