# Supplementary material for: RIN3 Is a Negative Regulator of Mast Cell Responses to SCF
Source: PLoS One. 2012 Nov 20;7(11):e49615. doi: 10.1371/journal.pone.0049615 (PMC3502454; doi:10.1371/journal.pone.0049615)
Supplement: Figure S2 — RIN3 silencing does not affect degranulation. The amount of granule release was measured in cells with control (white bars) or knock down levels of RIN3 (gray bars). All cells were primed with biotinylated IgE and then incubated with indicated concentrations of streptavidin (antigen). Percent granule release was calculated as β-hexosaminidase activity: supernatant/(supernatant + lysate). Graph is compilation of two independent experiments performed in triplicate. (PDF) [file pone.0049615.s002.pdf]

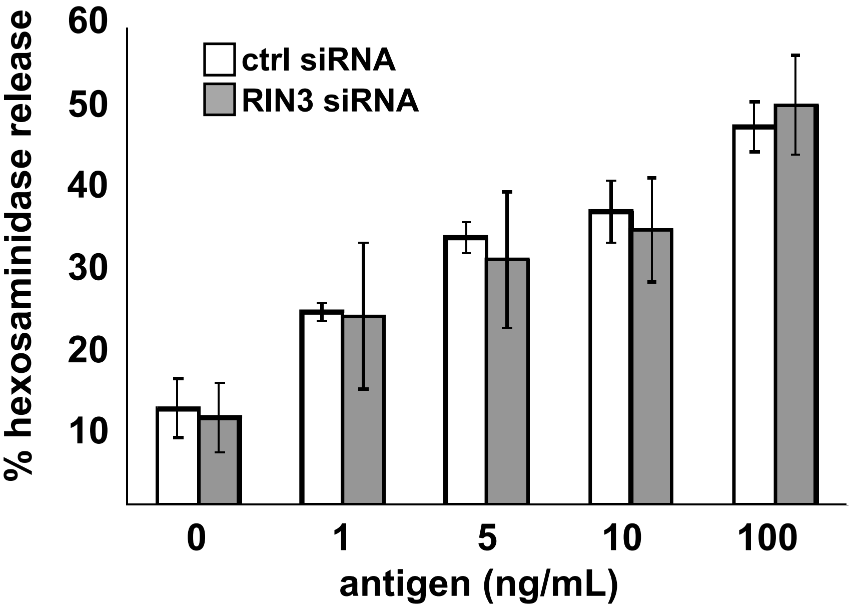

Figure S2. *RIN3 silencing does not affect degranulation.*
The amount of granule release was measured in cells with control (white bars) or knock down levels of RIN3 (gray bars). All cells were primed with biotinylated IgE and then incubated with indicated concentrations of streptavidin (antigen). Percent granule release was calculated as β-hexosaminidase activity: supernatant/(supernatant + lysate). Graph is compilation of two independent experiments performed in triplicate.
